# *Anopheles* larval control deserves a greater priority: lessons in malaria elimination from China

**DOI:** 10.1136/bmj-2024-080662

**Published:** 2025-04-22

**Authors:** Xinyue Fang, Liping Yang, Dongsheng Ren, Simon I Hay, Haoqiang Ji, Yelin Sun, Chunchun Zhao, Qiyong Liu

**Affiliations:** 1 National Key Laboratory of Intelligent Tracking and Forecasting for Infectious Diseases, National Institute for Communicable Disease Control and Prevention, Chinese Center for Disease Control and Prevention; WHO Collaborating Centre for Vector Surveillance and Management, Beijing, China; 2 Department of Vector Control, School of Public Health, Cheeloo College of Medicine, Shandong University, Jinan, China; 3 Department of Health Metrics Sciences, School of Medicine, University of Washington, Seattle, WA, USA

## Abstract

**Qiyong Liu and colleagues** review China’s integrated approaches and proactive practices for controlling Anopheles larvae, offering valuable insights for other countries striving to control or eliminate malaria

Malaria remains a major global public health issue. In 2022, there were 249 million malaria cases worldwide, an increase of five million from 2021.^1^ Since 2015, the annual number of cases has risen steadily, with the greatest surge of 11 million cases occurring between 2019 and 2020.^1^ As of 2022, malaria remains endemic in 85 countries, including the territory of French Guiana.^1^ China, once heavily affected by malaria, achieved a milestone in 2021 when the World Health Organization certified it as malaria-free.^2^ ^3^ Sustainable management of vectors, including larval sources, was a key factor contributing to the country’s success in eliminating malaria.

Malaria is a life threatening disease caused by *Plasmodium* through bites from infected female *Anopheles* mosquitoes, the main vectors of malaria. Mosquito control is therefore a core component of the malaria elimination strategy.^2^ The extensive application of insecticides has played an important part in controlling mosquitoes. In 1955, WHO adopted indoor residual spraying as the core vector control intervention targeting adult mosquitoes under the Global Malaria Eradication Programme.^4^ ^5^ However, killing the adult mosquitoes provides only temporary palliation without tackling the main mosquito breeding sources. Larval control, on the other hand, focuses on targeting the immature, aquatic stages of mosquitoes, preventing them from developing into adults. This approach reduces adult mosquito populations and ultimately contributes to effective malaria control.^4^

In China, a number of proactive approaches were implemented to control mosquito larvae. These included the application of larvicides and the removal of mosquito breeding habitats. Additionally, innovative agricultural practices—for example, altering irrigation patterns; rotating rice and upland crops such as cotton, wheat, and vegetables that resist drought and do not require regular irrigation; and co-cultivating rice and fish—were employed to reduce larval populations and suppress adult mosquito reproduction. Nationwide health campaigns and urbanisation further strengthened efforts to eliminate mosquito breeding sites by improving residential environmental hygiene. Together, these integrated approaches disrupted the *Anopheles* mosquito life cycle, reduced the adult population density, and contributed to China’s success in malaria elimination.

## Application of larvicides in malaria epidemic regions

In the comprehensive management of mosquito borne diseases, use of insecticides remains the primary and most direct method of mosquito control, especially during the peak mosquito season.^5^ For example, hexachlorocyclohexane and dipterex were widely applied as the main insecticides to reduce larval mosquito density.^6^ However, the extensive use of chemical larvicides led to serious environmental concerns such as toxic residues in water and soil. Reports indicated that the organochlorine level in the Han river reached 1075 ng/L, more than double the EU limit of 500 ng/L.^7^ Additionally, despite being banned for over 20 years, DDT (dichlorodiphenyltrichloroethane) residues were still detectable in soils across the Yangtze river delta, particularly in paddy fields.^8^ Another consequence of extensive pesticide use was the development of resistance. A review found that resistance to DDT among *Anopheles sinensis* was ubiquitous during the 1990s, with 89% of 27 surveillance sites across five Chinese provinces exhibiting significant or emerging resistance.^9^

In contrast to chemical insecticides, biolarvicides such as *Bacillus sphaericus* and *Bacillus thuringiensis israelensis* offer a more environmentally friendly alternative for larval control because of their host specificity and minimal ecological impact.^10^ Studies have showed that *B sphaericus* reduced mosquito fecundity and suppressed mosquito population density to a low level.^11^ For example, the application of *B sphaericus* and *B thuringiensis israelensis* decreased the larval density of *An sinensis* by 75-100% and 93-99%, respectively.^12^ ^13^ Optimal insecticidal effects were achieved by targeting younger larvae with lower concentrations of *B thuringiensis israelensis*, providing a more resource efficient and environmentally friendly approach.^14^ Recent adoption of drone technology has further enhanced the larviciding programme.^15^ In Zhejiang province, the first round of drone spraying reduced mosquito population density by 27%.^16^ Drone spraying has also been used in Africa, where it has shown promising results in controlling malaria.^15^

## Innovations in agricultural practices

In addition to larvicidal applications, removing mosquito breeding sites has proved an effective and sustainable intervention for mosquito control. Mosquito larvae develop in stagnant water, and paddy fields are often prime breeding grounds for *Anopheles* mosquitoes. Moreover, the vegetation surrounding paddy fields creates a protected environment for mosquito larvae to hatch and develop. Traditional flooding irrigation methods exacerbate the problem by creating stagnant water in rice fields, offering ideal conditions for mosquito breeding.

Innovative approaches such as intermittent, moistening, and controlled irrigation, along with planting rotations and rice-fish co-culture, have shown considerable success in reducing *Anopheles* breeding sites and larval densities. These methods not only have long term impacts on mosquito control but are also cost effective, enhancing their applicability in low income regions or areas with extensive cultivation that may inadvertently support mosquito populations.

### Intermittent irrigation

Intermittent irrigation refers to alternating periods of irrigating a field and then allowing it to dry, either actively or passively, based on the rice plant’s growth stage and its sensitivity to water stress. In the early 1930s, intermittent irrigation was successfully implemented in countries such as India and Japan, with experiments showing its effectiveness in controlling mosquitoes breeding in paddy fields.^5^ Since 1949, with the initiation of government health campaigns and prioritisation of vector borne disease control in China, research on intermittent irrigation has expanded to evaluate its impact on larval growth and oviposition by female mosquitoes. Hou C et al found that intermittent irrigation significantly altered the ecological environment of paddy fields, resulting in a 74% reduction in the density of *Anopheles* larvae, an increase in rice yields by 338 kg per hectare, and a saving of over 2800 m^3^ of irrigation water.^17^

### Moistening irrigation

Moistening irrigation is a water saving irrigation method that uses shallow water irrigation to prevent the persistence of standing water in paddy fields. After the rice seedlings establish themselves, shallow irrigation water is provided based on the crop’s growth requirements, with subsequent irrigation occurring only after the previous water has dried completely. Unlike traditional pond or flood irrigation, moistening irrigation reduces the volume of standing water and thus decreases mosquito breeding sites. In addition to its role in mosquito control, this method conserves water resources and increases crop yields. An experiment undertaken in the Yellow river irrigation area in Henan province showed that moistening irrigation significantly reduced the density of *An sinensis* in rice fields.^18^ Compared with flooded paddy fields, larval density decreased by 84-86%, while rice yields increased by about 10%.^19^

### Controlled irrigation

Controlled irrigation maintains a thin layer of water (10-30 mm) over the field after rice seedlings are transplanted. The timing and amount of irrigation are determined by monitoring root-soil moisture, ensuring that water does not persist in the field after irrigation.^20^ This method is particularly suited to low lying fields with high groundwater levels prone to waterlogging, as well as cold, waterlogged paddy fields in hilly and mountainous areas, wetlands, and polder fields in river plains.

**Fig 1:**
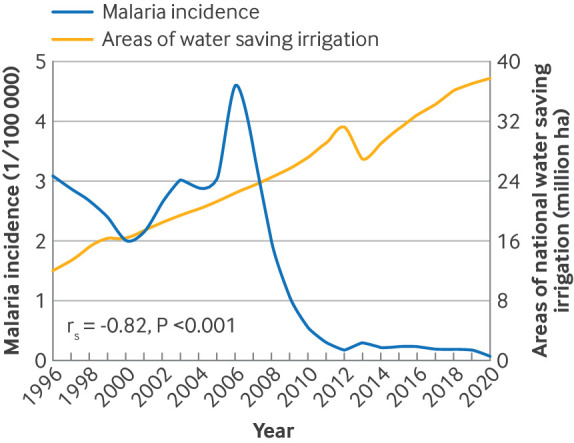
Relation between malaria incidence and national water saving irrigation area from 1996 to 2020

Initially implemented in countries such as India and Japan, intermittent irrigation was later introduced into China and adapted to local environmental conditions. Adoption of these irrigation techniques followed extensive experimentation, based on the introduction and absorption of irrigation technologies in other countries, incorporating lessons from international practices, while tailoring them to meet local needs. All three methods—intermittent, moistening, and controlled irrigation—showed remarkable effectiveness in managing mosquito larval breeding, conserving water, and increasing crop yields. A significant negative correlation was found between the area of water saving irrigation practices and malaria incidence at the national level in China (fig 1). Selection of the most suitable irrigation method, however, depends on factors such as the local climate, soil conditions, and available irrigation facilities.

### Paddy-upland rotation

The tillage pattern entails alternating rice cultivation in the summer with dry crops, such as wheat or vegetables, in the winter. Traditionally, paddy fields were left fallow during the cold winter months. By planting dry crops such as wheat, corn, cotton, and vegetables instead, the land—referred to as “upland”—is used more efficiently.^21^ This approach reduces considerably the overwintering mosquito larvae in paddy fields. Sichuan province implemented a paddy-upland rotation system that reduced the area of flooded paddy fields by more than half, from 967 000 to 400 000 hectares between 1983 and 1997.^21^ The absence of standing water in winter led to a reduction of over 90% in *An sinensis* larval density after the implementation of this rotation system, contributing to a significant decline in malaria incidence.^21^ Data from 1994-99 indicated that paddy-upland rotation contributed to a 98% decrease in *An sinensis* density and an 84% decline in the annual incidence of malaria.^22^ This analysis also showed a significant negative association between the implementation of paddy-upland rotation and malaria incidence.^22^

### Rice-fish co-culture

Rice-fish co-culture is a farming system that integrates rice planting with fish farming. This practice has a long history in China, with records dating back to 220-280 AD. Rice-fish co-culture was initially aimed at enhancing agricultural production.^5^ By the 1930s, rice-fish co-culture was proposed as a method for mosquito control. Commonly farmed fish species, such as *Cyprinus carpio* and *Ctenopharyngodon idella*, were widely promoted through various experiments across China.^5^ By 2009, roughly 10 million of China’s 24.4 million hectares of paddy fields were used for fish farming.^23^ Studies showed that rice-fish co-culture reduced the density of *Anopheles* larvae in paddy fields by 80% compared with fields without fish.^24^ Additionally, a study in Jiangsu province reported that rice-fish co-culture generated additional income through the sale of fish.^23^ Thus, beyond controlling *An sinensis* breeding, rice-fish co-culture also contributed to improved rice production and economic benefits.

## Improvement of residential hygiene

Improving residential environments is a critical component of mosquito breeding site management. Since the 1950s, national health campaigns have focused on managing the primary habitats of mosquitoes, such as ditches and ponds, to disrupt their reproductive cycles. These efforts have contributed to improving community hygiene and the control of infectious diseases, including malaria.^3^ The removal of mosquito breeding sites and the implementation of mosquito prevention and control measures have been incorporated into a series of hygiene and health promotion programmes, such as the establishment and recognition of hygienic villages, towns, and cities across China.^25^ In Zhejiang province, for example, breeding site management practices led to a significant decrease in the presence of larvae, from 23.98% to 2.56% in 2008.^26^ Another pilot project in Zhejiang was launched to create “mosquito-free villages” by using environmentally friendly methods to reduce mosquito density.^27^ These results were consistent with findings from a study in Anting New Town, Shanghai, where *Anopheles* larval control strategies were integrated into urban planning, including the reconstruction of landscape water bodies to prevent larval breeding by ensuring water flow.^28^

**Fig 2:**
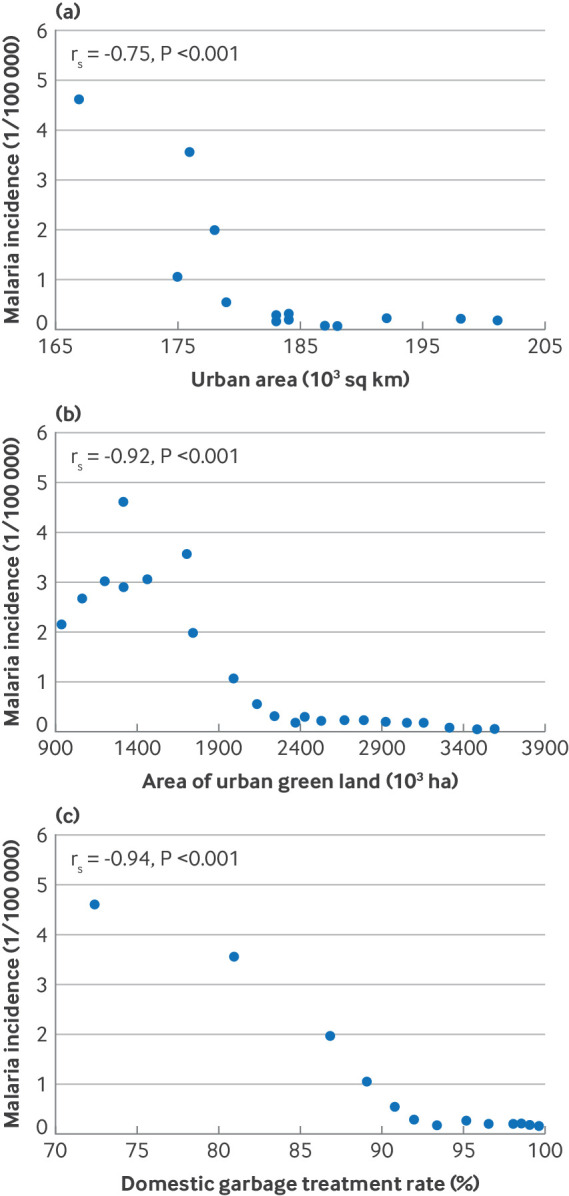
Relation between malaria incidence and residential environment in China: (a) urban area 2006-21 (b) area of urban green land 2001-22, and (c) domestic garbage treatment rate 2006-22

Over the past few decades, China has experienced a massive rural-to-urban migration, leading to the conversion of vast areas of farmland into urban developments.^29^ The expansion of urban buildings and infrastructure, including parks, green spaces, and improved waste management systems, has enhanced the residential environment,^29^ playing a vital part in reducing the incidence of malaria (fig 2). While urbanisation may not always lead to immediate improvements in residential environments, particularly in terms of eliminating mosquito breeding habitats, it undoubtedly bestows considerable advantages.

Overall, efforts to improve residential environmental hygiene through national health campaigns and urbanisation in China have been effective in removing mosquito breeding sites. These interventions have contributed to a significant reduction in malaria transmission by tackling key factors that support mosquito breeding.

## Integrated One Health principle in malaria elimination

It is increasingly recognised that the health of humans, animals, and the environment are interconnected and form an inseparable system.^30^ Malaria elimination cannot be achieved by a single discipline or sector alone, underscoring the importance of the One Health principle in controlling and eliminating malaria.

**Fig 3:**
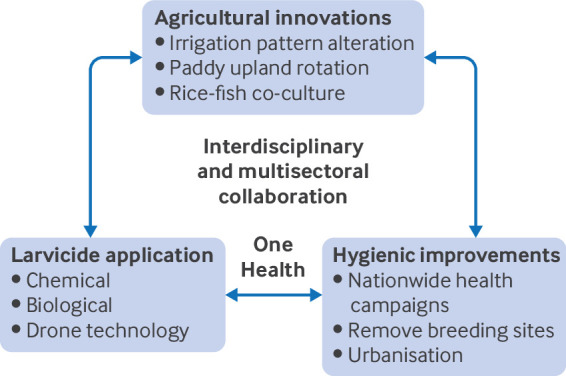
Integrated One Health principle in *Anopheles* larval control for malaria elimination

One Health is a globally recognised health strategy based on the premise that human health is closely interconnected with the health of animals and our shared environment.^30^ It embraces collaboration among a diverse range of experts working across multiple disciplines and departments to enhance the health of people, animals, and the environment.^30^ Here, we summarise China’s experiences with integrating *Anopheles* larval control into the One Health principle—illustrated in figure 3—which could serve as a model for other countries in their efforts to combat malaria.

*Agricultural innovations*—Paddy fields provide an ideal environment for *Anopheles* larval breeding, which emphasises the importance of integrated agricultural approaches. Combining agricultural approaches with One Health would not only reduce larval density but also benefit the environment and animals. Different irrigation methods are conducive to maintaining soil fertility in a more balanced way and using water resources efficiently, which could protect the ecological integrity of rice fields. Also, the benefits of these methods by saving water and increasing yield make them particularly applicable to low income countries and regions. Paddy upland rotation and rice-fish co-culture also contribute to building a sustainable and balanced ecosystem and supporting biodiversity in the process.

*Environmental improvement*—The improvement of residential environments is a crucial component that sustainably balances and optimises the health of humans, animals, and ecosystems. China has initiated a universal health campaign and community level health promotion to remove larval breeding sites and provide better access to health services and improved sanitation. These efforts have contributed to a reduced risk of malaria and improved human health. Additionally, efforts to improve community environmental hygiene can help protect animals from mosquito bites.

*Larvicide application*—The efficacy of biolarvicides offers an environmentally friendly alternative to chemical larvicides in pursuing malaria control within the One Health framework. To prevent environmental contamination and resistance resulting from the extensive application of chemical insecticides, China has promoted the use of biolarvicides. These biolarvicides effectively reduced larval density while preserving biodiversity and protecting the environment.

*Interdisciplinary and multisectoral collaboration*—Malaria elimination requires collaboration among a panel of experts working across agriculture, fishery, chemistry, biology, ecology, and medicine. Multiple disciplines are interrelated and coordinated to achieve mosquito control comprehensively and sustainably, promoting the health of humans, animals, and the environment. Efforts made by joint involvement with the government, the community, and the public have contributed appreciably to the successful elimination of malaria.

## Conclusion

Malaria remains a considerable threat to public health, with many countries still grappling with endemic cases. In China, control of *Anopheles* larvae has played an essential part in reducing the incidence of malaria as the country progressed from malaria control to elimination. Through constantly exploring innovative approaches, the combined applications of innovative irrigation methods, residential environment improvements, and biolarvicides have proved effective in controlling mosquito larvae. These methods, aligned with the concept of One Health*,* have made an important contribution to malaria elimination in China. The methods may not always be directly applicable in other countries or regions, and tailored methods are essential to tackle specific local ecological and epidemiological conditions. The lessons learnt from China’s experience offer valuable insights for other countries or regions striving to eliminate malaria, accelerating the progress towards a malaria-free world.

**Key messages**

- *Anopheles* mosquitoes are main vectors of malaria globally and *Anopheles* larval control is critical in reducing the morbidity and mortality of malaria
- These practices, including agricultural innovations, environmental hygiene improvements, and optimised application of larvicides, provide useful references for other countries facing a heavy burden of malaria and pursing malaria control or elimination.
- Integrating *Anopheles* larval control with the *One Health* principle shows much potential for effective malaria control and elimination

## Notes

Contributors and sources: QYL, as the principal investigator of *Anopheles* control for malaria elimination and a chief scientist, has long been engaged in vector control, climate change, and health research, and has made outstanding contributions to vector control and infectious disease prevention and control. QYL designed the article and supervised the research planning. XYF and LPY (joint first authors) wrote the article. HQJ and YLS produced the figures. DSR and CCZ collected the literature and data. SH provided constructive suggestions on the draft manuscript. All authors read and approved the manuscript. The corresponding author confirmed that all authors have seen and approved the final text.

Provenance and peer review: Commissioned; externally peer reviewed.

This article is part of a collection developed in partnership with the Chinese Center for Disease Control and Prevention, Beijing. Open access fees were funded by the Gates Foundation, China. *The BMJ* commissioned, peer reviewed, edited, and made the decision to publish. Rachael Hinton and Jocalyn Clark were the lead editors for *The BMJ*.
